# Design of a Single-Layer ±45° Dual-Polarized Directional Array Antenna for Millimeter Wave Applications ^†^

**DOI:** 10.3390/s21134326

**Published:** 2021-06-24

**Authors:** Qinyi Lv, Yu-Hang Yang, Shi-Gang Zhou, Chan Shao, Deyun Zhou, Chow-Yen-Desmond Sim

**Affiliations:** 1 School of Electronics and Information, Northwestern Polytechnical University, Xi’an 710129, China; lvqinyi@nwpu.edu.cn (Q.L.); sgzhou@nwpu.edu.cn (S.-G.Z.); sc18700496153@163.com (C.S.); dyzhou@nwpu.edu.cn (D.Z.); 2 Department of Electrical Engineering, Feng Chia University, Taichung 40724, Taiwan; cysim@fcu.edu.tw

**Keywords:** millimeter wave (mm-wave), dual linearly polarized, dual circularly polarized (CP), single layer

## Abstract

A single-layer ±45° dual-polarized directional array antenna for millimeter wave (mm-wave) applications is designed in this communication. Based on the theory of orthogonal circularly polarized (CP) wave multiplexing, two ports of a series-fed dual CP array are fed with equal amplitudes, and the array can radiate a linearly polarized wave with ±45° polarization orientations through the adjustment of the feeding phase difference. As the two ports of the series-fed array are simultaneously excited, the antenna can achieve directional radiation. In addition, the cross-polarization level of the array can be effectively suppressed by placing two series-fed arrays side by side. A prototype of the designed array antenna operating at 30 GHz is fabricated and measured; the working bandwidth of the proposed antenna is approximately 3.5%. Owing to its simple structure and directional radiation, the proposed antenna array is a competitive candidate for mm-wave applications.

## 1. Introduction

Millimeter wave (mm-wave) technology has many attractive application scenarios, such as gesture radars, automotive radars, imaging sensors, and the rapidly developing fifth-generation mobile communication system [1,2,3,4]. The antenna array is an important part of the mm-wave system, and the balance between its performance, cost, size, and integration difficulty has an important impact on the further promotion of mm-wave applications.

The mm-wave antenna printed on a single-layer substrate is an extremely competitive candidate due to its huge advantages in integration difficulty and cost. For example, the key challenge in mm-wave antenna-in-package (AiP) technology is how to minimize the interconnect loss between the die and antenna [5]. However, for the single-layer antenna, the antenna array, feeding network, and die can be printed on a layer substrate to reduce the interconnect loss. Even in some AiP packaging technology, only one layer of copper can be used to design antennas, such as the embedded wafer-level ball grid array (eWLB) technology proposed in [6]. For mm-wave systems based on printed circuit board (PCB) technology, single-layer antennas also have strong application requirements. In the design, development, and processing of a mm-wave radar reported by the company TI [7], the whole development board is composed of multi-layer substrates bonded using a prepreg film. The top high-frequency substrate is used to print the radio frequency (RF) circuit and antenna array and to arrange the RF chip. The bottom FR4 substrates are used to print the digital circuit. Therefore, the antenna elements and feeding network should be designed and processed on a single-layer substrate.

The most common single-layer antenna arrays are patch antenna and slot antenna. The arrays in [8,9,10] are all series-fed arrays fed by microstrip lines. This type of antenna is also the most commonly used mm-wave antenna array in engineering practice. With the development of substrate integrated circuits, the substrate integrated waveguide (SIW) slot arrays have been widely studied because of their compact and lightweight structure [11,12,13]. In addition, grid antenna arrays printed on single-layer substrate are also widely used in mm-wave radar [14,15,16,17], as these antennas have the advantages of wide impedance bandwidth, high gain, and high polarization purity.

Dual-polarized antennas are widely used in modern wireless communication systems owing to their numerous advantages such as increased channel capacity and anti-multipath fading [18]. Based on LTCC technology, the mm-wave antenna array designed in [19,20] achieves wideband dual-polarized radiation, and the radiation pattern within the bandwidth remains stable. In addition, the dual-polarized antenna array designed by multilayer PCB process in [21] achieves 50% wideband radiation. Although the antenna in [19,20,21] achieves excellent wideband dual-polarized radiation, in many applications, we expect the antenna array to be printed on a single-layer substrate. In a dual-polarized planar antenna printed on a single-layer substrate [22], ±45° dual-polarized series-fed arrays based on SIW share the aperture in an interdigital shape. A single-layer dual-polarized cross-slot planar array antenna with high port isolation is presented in [23]. All the antennas in [22,23] adopt series-fed subarrays, and thus, the maximum radiation direction of each array shifts with frequency changes; such behavior is not expected in several applications. In [24], an 8 × 8 dual-polarized traveling-wave antenna array is proposed. Thanks to the differential-series-fed method, the maximum radiation direction of the antenna keeps stable within the operation bandwidth, but resistors need to be set in the array to absorb the remaining energy of the series-fed array.

The existing design idea of an mm-wave dual-polarized array still follows the design method of the Sub-6 GHz array, that is, two orthogonal radiation elements are placed in the limited physical space [25]. Obviously, in the mm-wave frequency band, it is difficult to design a dual-polarized array on a very low-profile one-dimensional structure. There are two feasible schemes: one is the interdigital structure used in [22] and the other is the cross-slot structure used in [23,24]. Both of these schemes have great limitations.

In this paper, a single-layer ±45° dual-polarized directional array antenna based on the theory of orthogonal CP wave multiplexing is designed. The proposed antenna does not require changes in polarization orientation through the adjustment of the physical directions of radiation elements. It achieves polarization adjustment by changing the phase difference of port feeding. This capability is beneficial for mm-wave applications with limited physical space; however, they require a large number of RF chips that can adjust the feeding phase. The radiation elements used in the proposed array have been introduced in detail in [26,27]. It should be pointed out that the proposed array uses the element designed in our previous work, just like the single-layer mm-wave arrays [8,9,10,11,12,13,14,15,16,17,18,19,20,21,22,23,24] use patch, slot, or grid elements. This work has been preliminary introduced in our conference paper [28].

The paper is organized as follows. In Section 2, the theory of orthogonal CP wave multiplexing is analyzed and introduced in detail. In Section 3, the design of the dual-polarized array is described, and a cross-polarization suppression method is presented. In Section 4, the measured results of the proposed array are discussed. In Section 5, relevant conclusions are derived.

## 2. Theoretical Analysis

The standard linearly polarized wave and CP wave can be regarded as two special elliptical polarized waves with an axial ratio equal to infinity and 0 dB. Generally, two orthogonal linearly polarized components are used as the orthogonal basis to synthesize arbitrarily polarized waves. For example, SR technology [29], which synthesizes CP wave using linearly polarized elements by changing the feeding phase of the elements, has been widely used in the design of CP array antennas. In this work, we use the left-hand CP (LHCP) and right-hand CP (RHCP) waves as an orthogonal basis in synthesizing the required linearly polarized waves.

**Figure 1:**
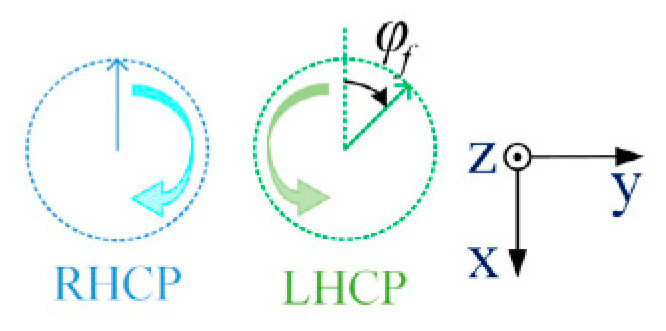
Ideal RHCP and LHCP components.

As shown in Figure 1, the ideal RHCP and LHCP components with respective amplitudes of $\left|{\vec{E}}_{R}\right|$ and $\left|{\vec{E}}_{L}\right|$ propagating in the $\hat{z}$ direction can be expressed as follows:

(1) $${\vec{E}}_{\mathrm{RHCP}}=\left|{\vec{E}}_{R}\right|\left(\hat{x}\cos\omega t+\hat{y}\sin\omega t\right)\;{\vec{E}}_{\mathrm{LHCP}}=\left|{\vec{E}}_{L}\right|\left(\hat{x}\cos\omega t-\hat{y}\sin\omega t\right)$$

Assume that the feeding phase difference between the two components is $\varphi_{f}$, and that the amplitudes of the RHCP and LHCP components are equal. The synthesized components can then be expressed as follows:

(2) $${\vec{E}}_{\mathrm{superposition}}={\vec{E}}_{\mathrm{RHCP}}+{\vec{E}}_{\mathrm{LHCP}}\;e^{j\varphi_{f}}\;=\left|{\vec{E}}_{R}\right|\left[\hat{x}\cos\omega t+\hat{y}\sin\omega t\right]+\left|{\vec{E}}_{L}\right|\left[\hat{x}\cos\left(\omega t+\varphi_{f}\right)-\hat{y}\sin\left(\omega t+\varphi_{f}\right)\right]\;=2\left|{\vec{E}}_{R}\right|\cos\frac{2\omega t+\varphi_{f}}{2}\left(\hat{x}\cos\frac{\varphi_{f}}{2}+\hat{y}\sin\frac{\varphi_{f}}{2}\right)$$

In Equation (2), when $\varphi_{f}=\frac{\pi }{2}$,

(3) $${\vec{E}}_{\mathrm{superposition}}=\sqrt{2}\left|{\vec{E}}_{R}\right|\cos\left(\omega t+\frac{\pi }{4}\right)\left(\hat{x}+\hat{y}\right)$$

When $\varphi_{f}=-\frac{\pi }{2}$,

(4) $${\vec{E}}_{\mathrm{superposition}}=\sqrt{2}\left|{\vec{E}}_{R}\right|\cos\left(\omega t-\frac{\pi }{4}\right)\left(\hat{x}-\hat{y}\right)$$

Equations (3) and (4) indicate that when the feeding phase difference between two orthogonal CP components is equal to 90° (i.e., the LHCP is 90° ahead of the RHCP), the superimposed field component is a linearly polarized wave at a 45° orientation. When the feeding phase difference is equal to −90° (i.e., the RHCP is 90° ahead of the LHCP), the superimposed field component is a linearly polarized wave at a −45° orientation.

## 3. ±45° Dual-Polarized Array Design

### 3.1. Array Design

**Figure 2:**
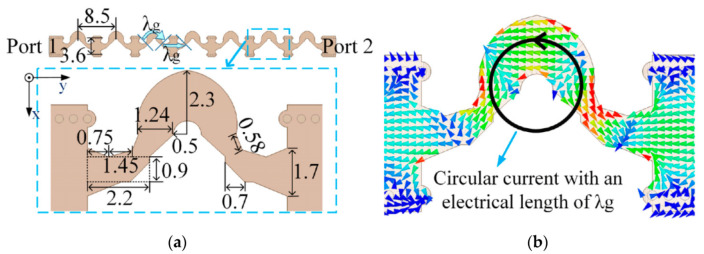
Radiation element and array. (**a**) Structures and dimensions of radiation element and array. (Unit: mm). (**b**) The current distributions on the curved microstrip line.

The structures and dimensions of the radiation element and array are shown in Figure 2, while the working principle is described in detail in [26,27]. When port 1 is excited, the array radiates LHCP waves; when port 2 is excited, the array radiates RHCP waves. The array adopts curved microstrip lines as CP radiation elements, the structure of which is a circle of a microstrip line with the electric length λg, and the current direction is similar to a ring. A circular current with an electrical length of λg can achieve CP radiation. An SIW is used as the feeding line between two radiation elements because it has lower radiation losses than microstrip line, and the electric lengths of SIW and microstrip line to SIW transition structure are equal to λg. Therefore, the feeding difference between adjacent radiation elements is 2*360° at 30 GHz, and the maximum radiation direction of the series-fed array points to the positive *z*-axis at 30 GHz. The proposed array is printed on Rogers RO4350B (tm) laminate (εr = 3.66, δ = 0.004, and thickness h = 20 mil).

**Figure 3:**
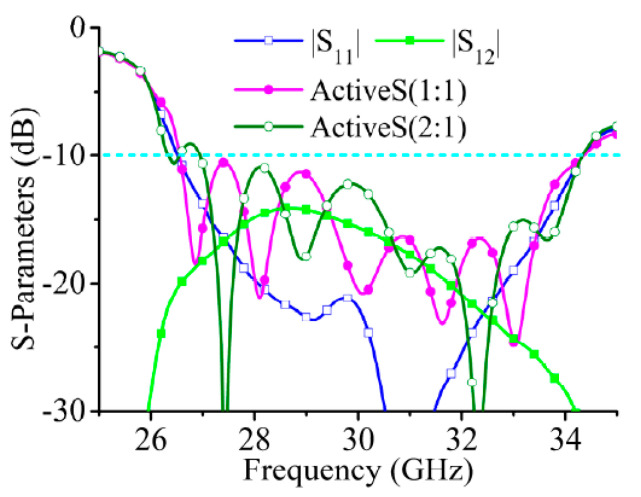
Simulated S-parameters of series-fed array. (To calculate active S-parameters, the feeding amplitudes, phases of port 1 and port 2 are set to 1 W, 0 deg and 1 W, 90 deg).

The simulated S-parameters are shown in Figure 3. As the two ports need to be excited simultaneously when the array is working, the isolation between the two ports influences impedance matching; thus, active S(1:1) and active S(2:1) are used here to express the impedance bandwidth of the array. In Figure 2, active S(1:1) and active S(2:1) are lower than −10 dB from 29 GHz to 31 GHz.

**Figure 4:**
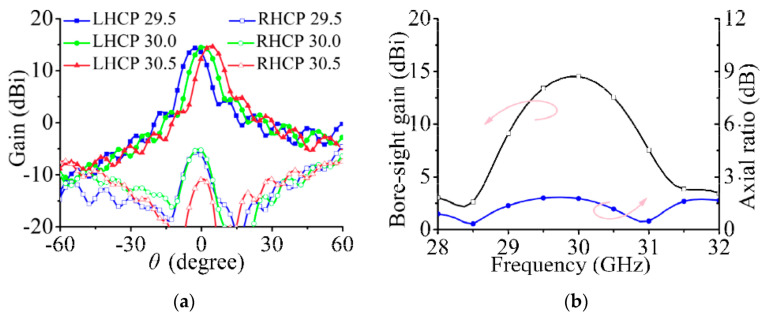
Simulated radiation performance of series-fed array only when port 1 is excited. (**a**) Radiation pattern in yoz plane at 29.5, 30, and 30.5 GHz. (**b**) Bore-sight gain and axial ratio.

The simulated radiation pattern in the yoz plane when port 1 is excited is shown in Figure 4a. At 29.5, 30, and 30.5 GHz, the maximum radiation directions of the array are −3°, 0°, and 3.3° and the maximum gains are 14.5, 14.7, and 14.6 dBi, respectively. The beam deflections at 29.5 and 30.5 GHz are caused by the characteristics of the series-fed array. The simulated gain and axial ratios at the positive *z*-axis direction are shown in Figure 4b.

**Figure 5:**
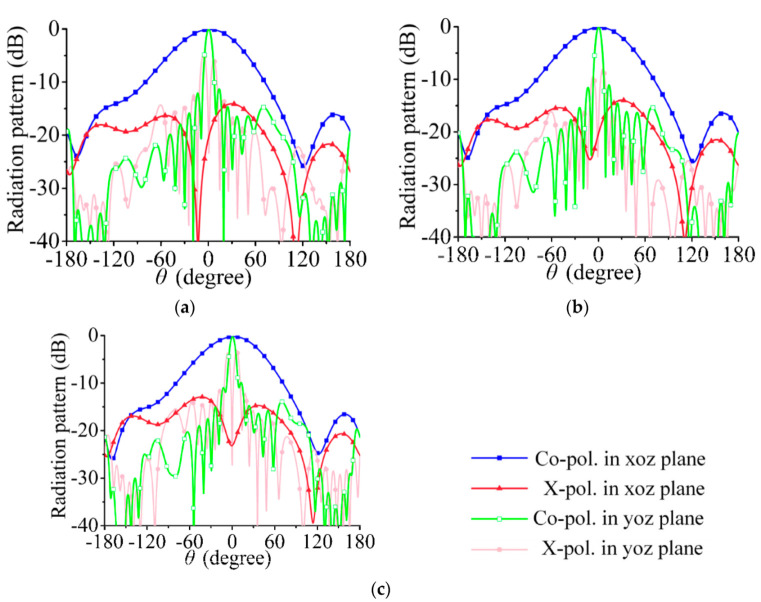
Simulated radiation pattern. (**a**) 29.5 GHz, (**b**) 30 GHz, (**c**) 30.5 GHz; co-polarized waves are −45° linearly polarized wave.

**Figure 6:**
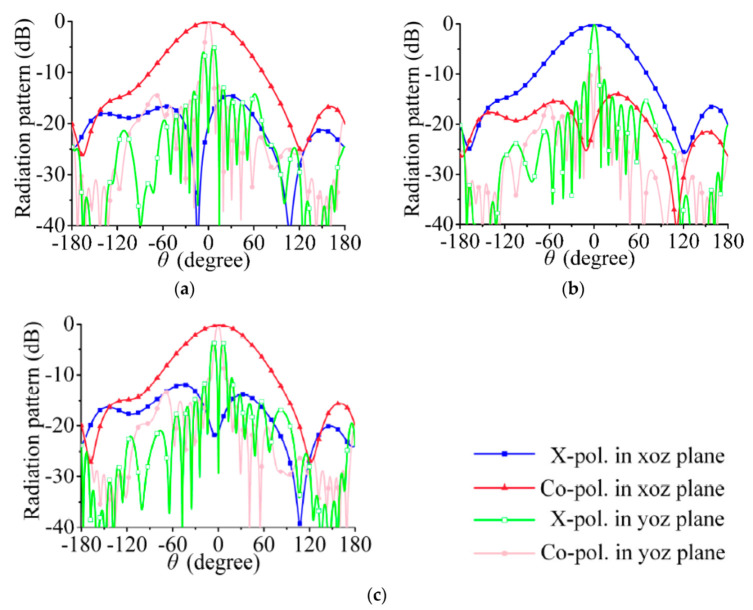
Simulated radiation pattern. (**a**) 29.5 GHz, (**b**) 30 GHz, (**c**) 30.5 GHz; co-polarized waves are 45° linearly polarized wave.

According to the above analysis, dual linearly polarized radiation can be achieved when port 1 and port 2 of the array in Figure 2 are excited at the same amplitude and the phase difference is ±90°. In Figure 5a–c, the feeding phases of port 1 and port 2 are 0° and 90°, respectively (i.e., the RHCP is 90° ahead of the LHCP), and the array radiates a −45° linearly polarized wave. In Figure 6a–c, the feeding phases of port 1 and port 2 are 0° and −90°, respectively (i.e., the LHCP is 90° ahead of the RHCP), and the co-polarization component is a 45° linearly polarized wave.

In Figure 5 and Figure 6, the maximum radiation direction of the array always points to the positive *z*-axis direction. In applications such as automotive radars, the stability of the beam direction of an antenna array within the working bandwidth greatly improves the detection accuracy of the whole machine.

**Figure 7:**
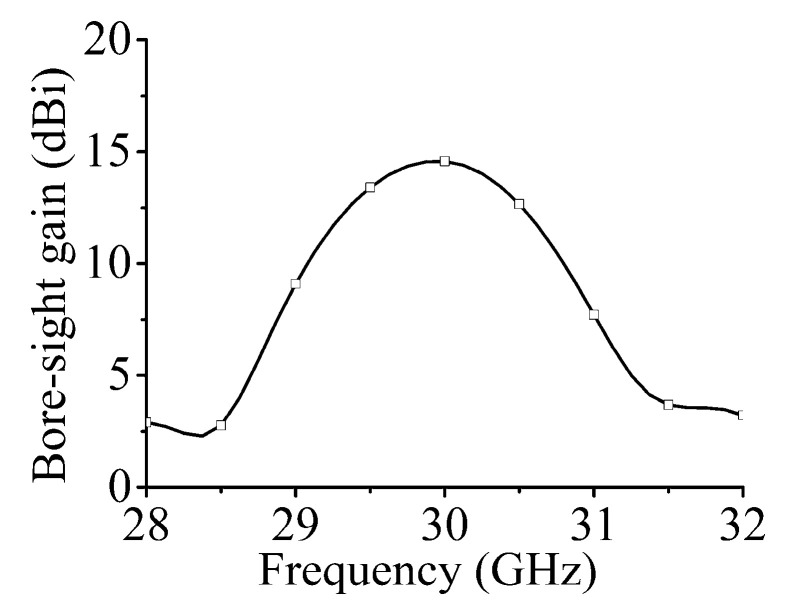
Bore-sight gains of dual-polarized array.

The bore-sight gains of the array in the positive *z*-axis direction are shown in Figure 7; the maximum gain at 30 GHz is 14.7 dBi. Although the beam direction of the array always points to the positive *z*-axis in the yoz plane, the variation trend of the gain with frequency is basically the same as that for the single port excited in Figure 4b. Hence, the 3-dB gain bandwidth of the array is the main factor limiting the operation bandwidth.

### 3.2. Causes of Cross-Polarization Deterioration

The cross-polarization levels in Figure 5 and Figure 6 are relatively high, and the co-polarization and cross-polarization levels are equal in the yoz plane when *θ* deviates 0°. In this section, the reasons behind the high level of cross-polarization are analyzed.

In Equation (2), when *φ_f_* is equal to 0° or 180°, the orientations of the superimposed field components are parallel to the y and x axes. When these two vectors are decomposed to ±45° orientations, the co-polarization and cross-polarization levels are equal.

**Figure 8:**
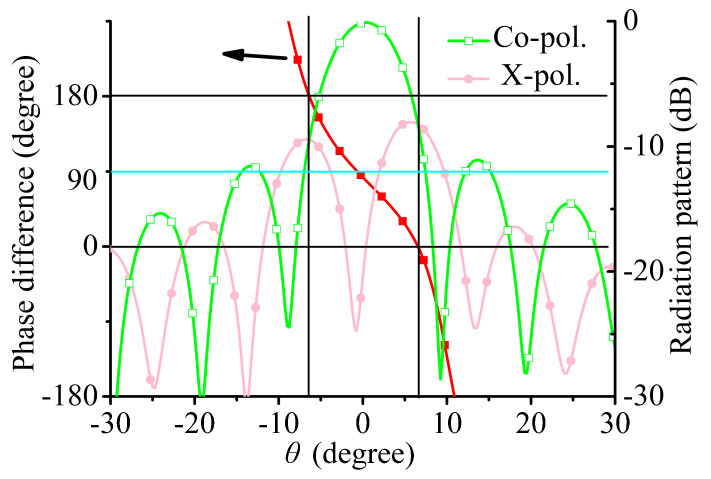
Phase difference between RHCP and LHCP waves at 30 GHz in the yoz plane, and the −45° linearly polarized radiation pattern at 30 GHz in the yoz plane.

Take 30 GHz as an example. The phase differences of RHCP and LHCP components in the yoz plane are shown in Figure 8, and it can be found that the *θ* angles where the co-polarization and cross-polarization levels are equal are the angles where the phase differences of RHCP and LHCP components are equal to 0° or 180°. In addition, when *θ* = 0°, the phase difference of the RHCP and LHCP components is not equal to 90°, mainly because of the limited purity of the CP wave.

**Figure 9:**
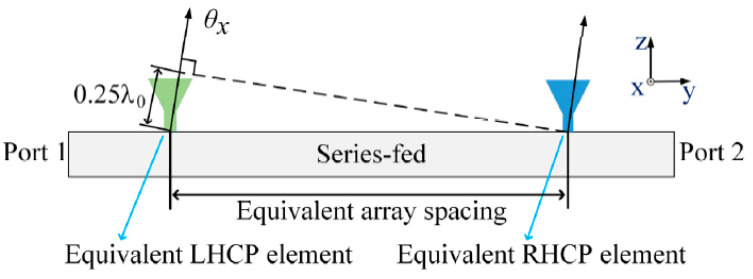
Equivalent array spacing leading to the deterioration of array cross-polarization at *θ*_x_ angle.

The reasons for the phase difference between the RHCP and LHCP components are discussed below. The structures of the radiation elements in the series-fed array are the same. Thus, when port 1 is excited, the radiation elements near port 1 radiate most of the energy. When port 2 is excited, the elements near port 2 radiate most of the energy. Therefore, as shown in Figure 9, although the RHCP and LHCP waves are radiated by the same series-fed array, the phase centers of the two components are not in the same position. This situation can be attributed to an excessively large array spacing; the greater the number of elements in the series-fed array, the larger the equivalent array spacing will be. However, the equivalent array spacing cannot be reduced by reducing the number of elements as the isolation between port 1 and port 2 will worsen.

### 3.3. Improvement of Polarization Purity

The analysis in the previous section indicates that the cross-polarization deterioration is caused by the excessively large equivalent array spacing of the RHCP and LHCP components. This section proposes a scheme to improve polarization purity.

**Figure 10:**
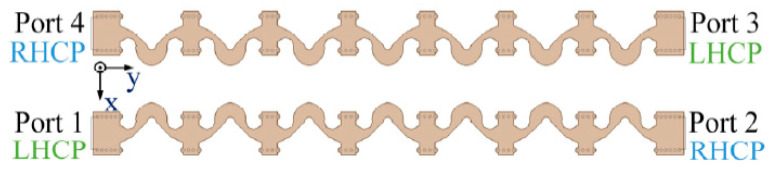
New array arrangement.

The new array arrangement is shown in Figure 10. A series-fed array is added above the original series-fed array, and the two arrays are mirrored along the *y*-axis. Improving the polarization purity only on a series-fed array is difficult. Therefore, a new series-fed array is added on the basis of the original array. Figure 10 shows two series-fed arrays and four ports. It also marks the rotation orientations of the CP wave radiated by the array when each port is separately excited.

The principle of improving the polarization purity of the new array is to introduce a new series-fed array, which is relatively close to the original series-fed array, and to solve the problem of the high cross-polarization level of the original single array through proper feeding. The new feeding methods are shown in Table 1. Take a −45° linearly polarized wave as an example. The phase difference between port 1 and port 2 has been analyzed in detail. In this section, the phase relationship between port 1 and port 4 is mainly studied. The CP orientations are the same when port 2 and port 4 are excited, but their feeding phases are 90° and −90°, respectively. This result is mainly due to the fact that the two series-fed arrays form a mirror relationship along the *y*-axis, resulting in a 180° phase difference in the CP waves when port 1 and port 4 are excited. Hence, the phase difference caused by the array placement direction needs to be compensated back by the feeding phase.

**Figure 11:**
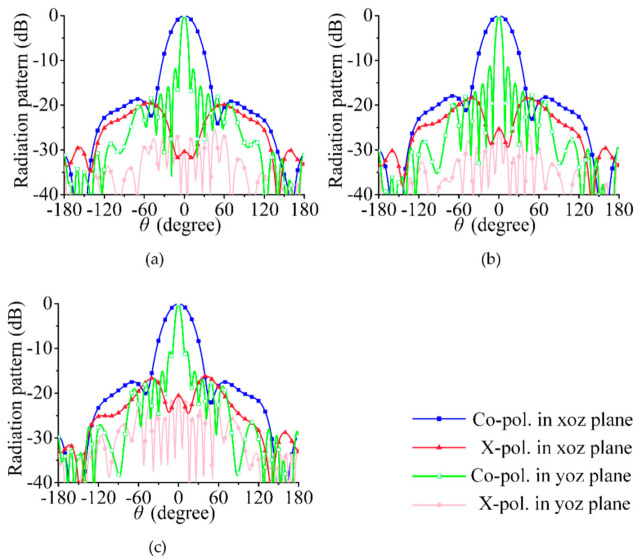
Simulated radiation pattern. (**a**) 29.5 GHz, (**b**) 30 GHz, (**c**) 30.5 GHz; co-polarized waves are −45° linearly polarized wave.

**Figure 12:**
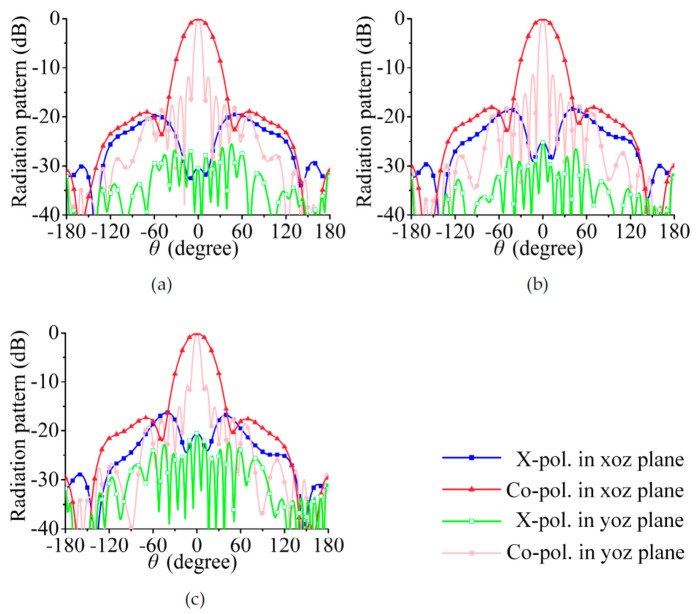
Simulated radiation pattern. (**a**) 29.5 GHz, (**b**) 30 GHz, (**c**) 30.5 GHz; co-polarized waves are 45° linearly polarized wave.

The simulated radiation patterns of the array are shown in Figure 11 and Figure 12. The beam width in the yoz plane is narrower than that in a single series-fed array. The 3-dB beam widths in Figure 5 are 72.0°, 73.1°, and 75.7°; those in Figure 11 are 34.7°, 34.9°, and 35.7°. The cross- polarization level in Figure 11 and Figure 12 is much lower than that in Figure 5 and Figure 6, especially in the yoz plane. This result shows that the cross-polarization suppression scheme proposed in this section is highly effective.

**Figure 13:**
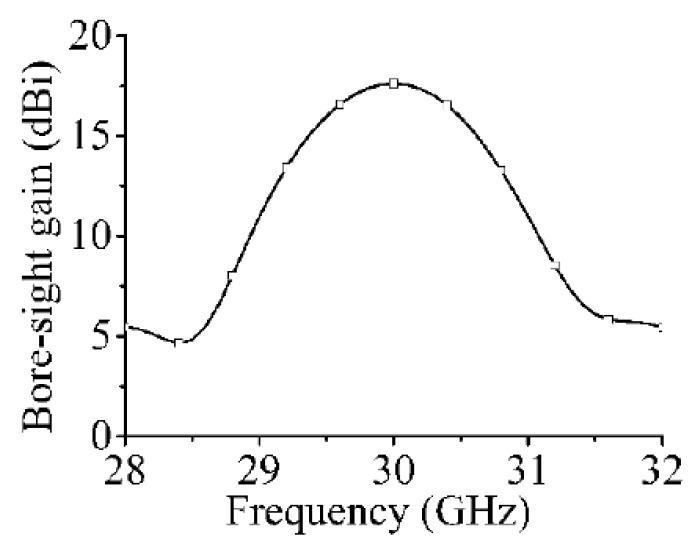
Bore-sight gains of dual-polarized array in Figure 9.

The simulated bore-sight gains of the array are shown in Figure 13. The maximum gain at 30 GHz is 17.6 dBi, which is 2.9 dBi higher than that of the single series-fed array in Figure 6.

## 4. Fabrication and Measurement Results

In a practical application, the four ports of the array in Figure 10 can be directly connected to RF chips to work normally. Limited by the experimental conditions, we need to design a feeding network which can provide the required phase relationship so as to verify the performance of the antenna. This measurement method is the same as that for differential-fed antennas in [30,31].

### 4.1. Feeding Work Design

**Figure 14:**
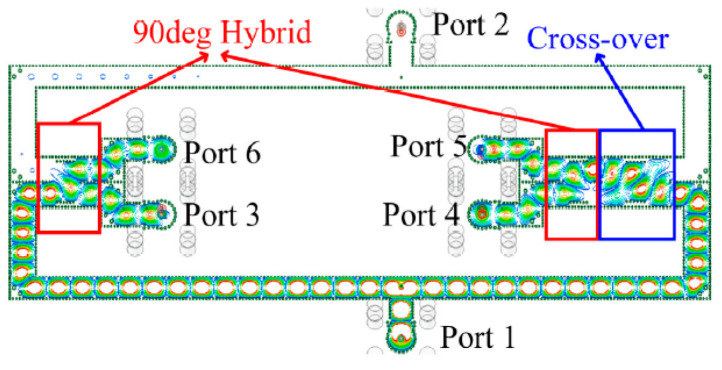
Structure of feeding network.

The structure of the feeding network is shown in Figure 14. Two 90° hybrids and a crossover are set up in the feeding network to produce the desired amplitude phase output. The feeding network is designed by SIW transmission line. In order to test the feed network, the SIW transmission line is connected to the coaxial port, and the interconnection structure is introduced in detail [26]. The design process of SIW 90° hybrid, and crossover is introduced in detail in [32]. In Figure 14, we find that the feed positions of port 1 and port 2 are not centered, which provides a 180° phase difference between ports 3, 6 and ports 4, 5. The 90° hybrid on the left can form a 90° phase difference between ports 3 and 6. With the 90° hybrid and crossover on the right, the four output ports can achieve equal amplitude output, and the phase difference between adjacent ports is 90° or −90°.

**Figure 15:**
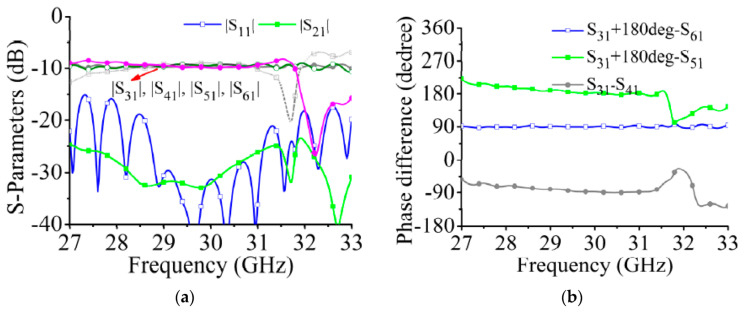
Simulated S-parameters of feeding network. (**a**) Output amplitudes. (**b**) Phase differences between adjacent ports. (The 180° phase difference caused by the different positions of the coaxial connectors of the output ports has been compensated).

To measure the S-parameters of the processed feeding network, we set the coaxial connectors of port 3 and port 4 above the substrate, and those of port 5 and port 6 below the substrate because the distance between port 3 and port 6 is too small and the installation space of the coaxial connectors is insufficient. This setup results in a 180° phase difference between ports 3 and 4 and ports 5 and 6; such phase differences are directly compensated in Figure 15.

The simulated S-parameters of the feeding network are shown in Figure 15. The four output ports achieve a constant amplitude output in the 29 to 31 GHz frequency band, and the insertion loss is approximately −9.5 dB. The phase differences of the output ports are shown in Figure 15b. When port 1 is excited, the phase relationships of the output ports satisfy the excitation phase required for −45° polarized radiation (Table 1). Considering the symmetry of the feeding network, we omit the results for when port 2 is excited.

**Figure 16:**
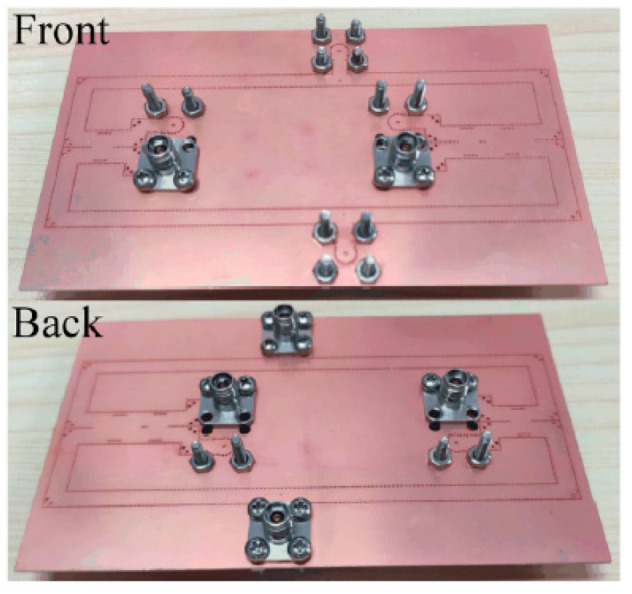
Photograph of feeding network used for radiation pattern measurement.

**Figure 17:**
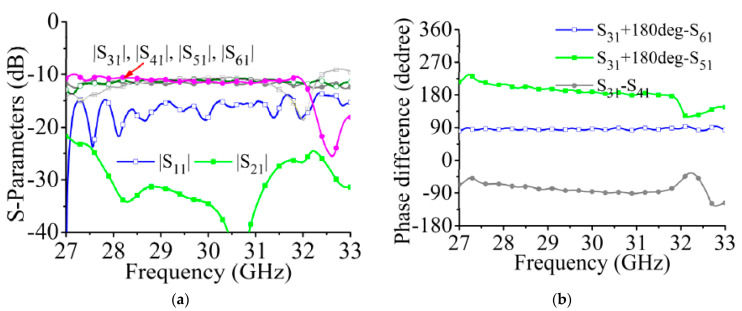
Measured S-parameters of the feeding network. (**a**) Output amplitudes. (**b**) Phase differences between adjacent ports.

The processed feeding network is shown in Figure 16. The measured S-parameters of the feeding network are shown in Figure 17. In the 29 to 31 GHz frequency band, the insertion loss is approximately −11.3 dB, which is 1.8 dB higher than the simulated result. This difference is mainly due to the roughness of the copper layer.

### 4.2. S-Parameters Measurement

**Figure 18:**
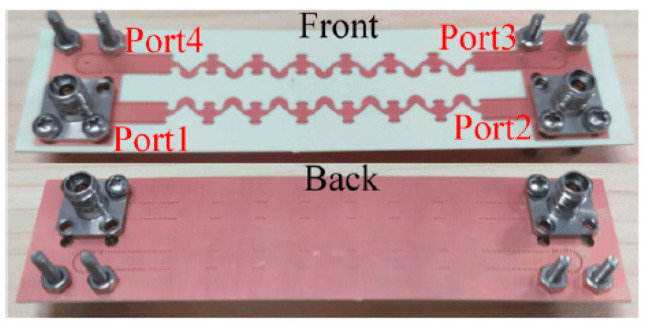
Photograph of array without a feeding network. In practical applications, the four ports are directly connected to the chip port with phase control function.

**Figure 19:**
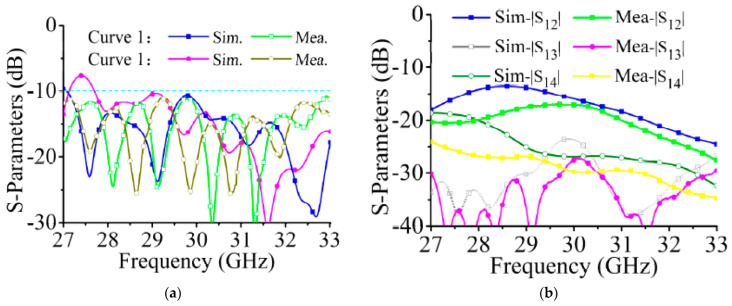
Measured and simulated S-parameters of the array without the feeding network. (**a**) Active S-parameters. (**b**) |S_12_|, |S_13_|, and |S_14_|.

The array without a feeding network is processed and shown in Figure 18. Four ports are connected to coaxial connectors. The measured and simulated S-parameters are shown in Figure 19. As the array uses a dual-port excitation mode of operation, active S-parameters are used to represent the impedance matching characteristics of the array. The expression of curve 1 is

(5) $$\mathrm{dB}(S_{11}+S_{12}\cdot e^{-j\frac{\pi }{2}}+S_{13}\cdot e^{-j\pi }+S_{14}\cdot e^{-j\frac{3\pi }{2}})$$

Curve 2:

(6) $$\mathrm{dB}(S_{11}+S_{12}\cdot e^{j\frac{\pi }{2}}+S_{13}\cdot e^{j\pi }+S_{14}\cdot e^{j\frac{3\pi }{2}})$$

In the calculation, the 180° phase difference of port 3 and port 4 caused by the coaxial position has been compensated. The measured results are lower than −10 dB from 29 GHz to 31 GHz. In Figure 19b, the isolations between adjacent arrays are greater than 20 dB.

### 4.3. Radiation Pattern Measurement

In this section, the feeding network in Figure 16 and the array in Figure 18 are connected to simulate and measure the radiation patterns of the dual-polarized array. It should be pointed out that the feeding network is only for testing the radiation pattern of the array. When the antenna array is working, the ports can be directly connected to the RF chips with the feed phase switching function to achieve polarization switching without the need for complicated feeding network.

**Figure 20:**
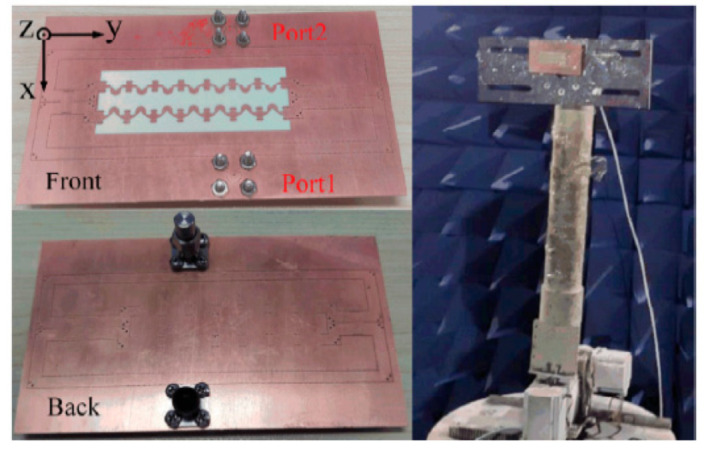
Photograph of the array for radiation pattern measurement.

**Figure 21:**
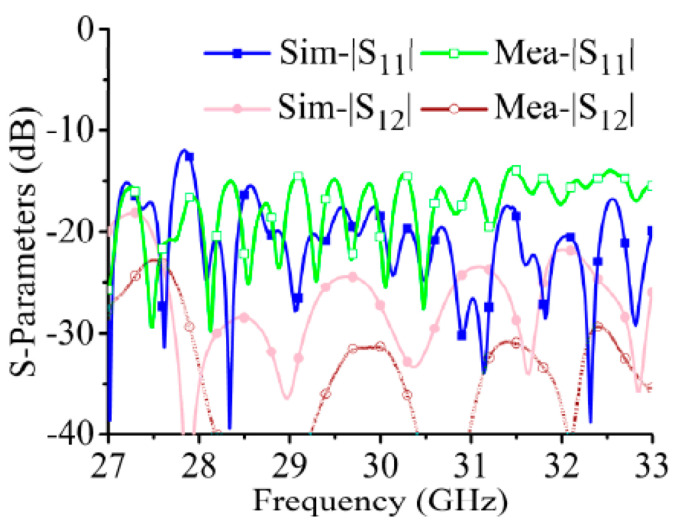
Measured and simulated S-parameters of the array.

The processed array structure is shown in Figure 20. The comparison of the simulated and measured S-parameters is shown in Figure 21. The measured |S_11_| are lower than –10 dB from 29 GHz to 30 GHz, and the measured port isolations are greater than 20 dB from 29 GHz to 20 GHz.

**Figure 22:**
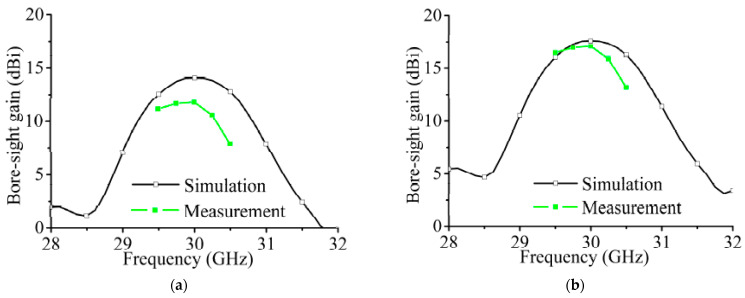
(**a**) The bore-sight gains of the array in Figure 18. (**b**) The calculated bore-sight gains after feeding network insertion loss compensation.

The comparison of the simulated and measured bore-sight gains is shown in Figure 22a. The simulated and measured maximum gains at 30 GHz are 14.2 and 11.8 dBi, respectively. To eliminate the influence of the feeding network on the array gain, we provide the array gains and insertion losses of the feeding network in Table 2. For the simulated results, the gain of the array without a feeding network can be estimated by the following equation:

(7) Gain_w(dB)=Gain_f(dB)+DL(dB)

*Gain_w* is the computed gain of the array without a feeding network, *Gain_f* is the gain of the array with a feeding network, and *DL* is the dielectric loss of the feeding network. In the above equation, the effect of the feeding network’s reflection on the computed gain is ignored, and only the effect of the insertion loss is considered.

For the simulated results in Table 2:

(8) Gain_w(dB)=Gain_f(dB)+DL(dB)=14.2 dB+(9.5 dB−6 dB)=17.7 dB

In (8), the dielectric loss of the feeding network is calculated by (9.5 dB–6 dB), where 9.5 dB is the insertion loss of the output port simulated in Figure 15a, and 6 dB is the insertion loss of the ideal lossless feeding network. The dielectric loss of the feeding network can be obtained by subtracting these two insertion losses. The gain calculated according to (8) is 0.1 dB higher than the gain of the simulation result in Figure 13, which shows the suitability of calculating the gain of the array without a feeding network according to (7). The calculation based on the measured results shows that the measured gain of the array at 30 GHz is about 17.28 dBi, which is lower than the simulated gain mainly because of the roughness of the copper layer. The measured and simulated gains after insertion loss compensation in the feeding network are shown in Figure 22b.

**Figure 23:**
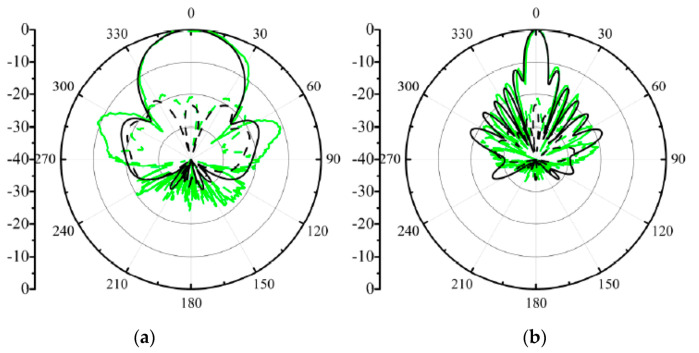

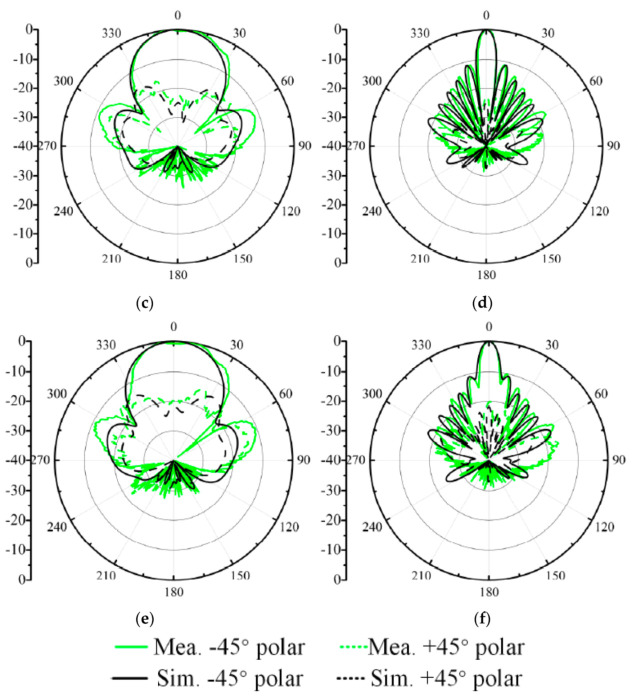
Measured and simulated radiation patterns of the proposed array when port 1 is excited; (**a**) 29.5 GHz at xoz plane; (**b**) 29.5 GHz at yoz plane; (**c**) 30 GHz at xoz plane; (**d**) 30 GHz at yoz plane; (**e**) 30.5 GHz at xoz plane; (**f**) 30.5 GHz at yoz plane.

The comparison of the measured and simulated radiation patterns at 29.5, 30, and 30.5 GHz is shown in Figure 23. Considering the symmetry of the array structure, we only provide the radiation patterns of the array excited by port 1. Relative to other series-fed arrays printed on single-layer substrates, the proposed dual-polarized array has a maximum radiation direction that does not change with the frequency and always points to the positive *z*-axis. The 3 dB beam widths in the xoz plane are approximately 8.3°, 7.8°, and 8.2° at three frequency points. The 3 dB beam widths in the yoz plane are approximately 36.8°, 37.4°, and 37.6°.

## 5. Conclusions

A single-layer ±45° dual-polarized directional array antenna using the radiation elements of a curved micro-strip line is designed in this paper, and the most prominent feature of the proposed array is that the maximum radiation direction remains stable with frequency changes. A method for improving polarization purity is proposed. The proposed array is then processed and measured. The measurement results are in good agreement with the simulation results. Thanks to the large number of applications of mm-wave chips, the proposed dual-polarized array is suitable for large-scale commercial applications of mm-wave radars.

## Figures and Tables

**Table 1:** Feeding phases of ports in Figure 10.

Feeding Phase	−45° Linearly Polarized Wave			45° Linearly Polarized Wave		
Port 1	0°	or	0°	0°	or	0°
Port 2	90°		90°	−90°		−90°
Port 3	180°		180°	180°		−180°
Port 4	−90°		270°	90°		−270°

**Table 2:** Array gain and feeding network isolation at 30 GHz.

	Array Gain with Feeding Network	Insertion Loss of Feeding Network	Array Gain without Feeding Network
Sim.	14.2 dBi (in Figure 22a)	9.5 dB (in Figure 15a)	17.6 dB (in Figure 13)
Mea.	11.8 dBi (in Figure 22a)	11.3 dB (in Figure 17a)	-
